# A Scoping Review on the Prevalence of Potentially Inappropriate Medication and Polypharmacy among Older People in a Lower-middle Income Country

**DOI:** 10.31662/jmaj.2025-0091

**Published:** 2025-08-08

**Authors:** Anjan Khadka, Arjun Poudel, Shakti Shrestha

**Affiliations:** 1 Department of Pharmacology, Nepalese Army Institute of Health Sciences, Kathmandu, Nepal; 2 School of Clinical Sciences, Queensland University of Technology, Brisbane, Australia; 3 School of Pharmacy and Pharmaceutical Sciences, The University of Queensland, Brisbane, Australia

**Keywords:** aged, drug utilization review, inappropriate prescribing, Nepal, polypharmacy, prevalence

## Abstract

There has been an increase in the number of older populations globally leading to a higher likelihood of multi-morbidities necessitating the use of multiple medications. This often poses the risk of potentially inappropriate medications (PIMs) and polypharmacy among older adults, but their data in the Nepalese context are lacking. This scoping review aimed to examine the published literature regarding the use of PIMs and polypharmacy in older people in Nepal. A literature search was conducted using three databases: PubMed, Embase, and Google Scholar. Articles published in English from 2011 to 2024 were retrieved for analysis to identify studies from Nepal, including the prevalence of PIMs and polypharmacy in older people aged ≥60 years. Studies were screened using Covidence (a web-based platform to perform reviews) and data were extracted using a specially designed form. The average values of the prevalence of PIMs and polypharmacy were derived from the selected studies. Seven articles involving 2341 older adults were included. The overall prevalence rates of PIMs and polypharmacy were 28.5% and 39.48%, respectively. Regional representation from Nepal included four studies from central Nepal and one each from western and eastern Nepal. The most common PIMs were benzodiazepines and non-steroidal anti-inflammatory drugs. PIM usage and polypharmacy are widely prevalent in Nepal. Hence, it is imperative to undertake effective measures to promote rational prescription practices for the geriatric population in Nepal.

## Introduction

The older adult population is increasing globally and so are the common health conditions associated with aging ^(1)^. In 2019, there were 1 billion older people aged ≥60 years worldwide, which is expected to increase by 40% by 2030 and double by 2050 ^(2)^. A similar trend has been predicted in the South Asian and Southeast Asian context ^(3), (4), (5)^. Nepal has seen an upsurge of the older population by 38.2% in 2021 following the previous census in 2011 of 2.97 million, making approximately one-tenth of the total population of Nepal ^(4)^. This increase indicates an annual growth rate of the older population by 3.5 times the average population growth rate ^(4)^.

The common tools to identify potentially inappropriate medications (PIMs) are Beers Criteria and the Screening Tool of Older Person’s Potentially Inappropriate Prescriptions (STOPP) ^(5), (6), (7)^. These tools include evidence-based recommendations on which drugs to avoid and guidance on which medications should be used cautiously as they may cause significant drug interactions or should be reduced based on renal function in elderly people ^(6), (7)^. While people are living longer, the aging phenomenon could cause a gradual decline in the physical and cognitive capacities of an individual ^(8)^. This could make older people more prone to suffer from multi-morbidities, particularly chronic illnesses that might necessitate the use of multiple medications ^(9), (10)^. The use of multiple medications increases the risk of inappropriate medication, drug-drug interactions, adverse drug responses, non-compliance, and increase in treatment costs ^(11), (12)^. Inappropriate medication use among older multi-morbid individuals is often associated with polypharmacy (use of ≥5 medicines) ^(11), (12), (13)^. Among various drugs used by older patients, the potential adverse risks of a certain medication may exceed the expected benefits; such drugs are referred to as PIMs ^(6), (13)^. The risk of continuing such medications in older adults often outweighs their benefits if appropriate action is not taken in time. This is because evidence suggests that PIMs and polypharmacy are often associated with poor health outcomes among older adults such as poor quality of life, increase in falls, frailty, hospitalization, disability, and mortality ^(10), (11), (12), (14)^. Global data from a recent meta-analysis suggest a high prevalence of PIMs (28.9%-43%) and polypharmacy (49.5%-62%) among older adults ^(15), (16)^. Although there is documented evidence of increasing PIM use and polypharmacy among older adults globally, there is a paucity of data from Nepal ^(9), (15), (16), (17), (18), (19)^. The concepts of PIMs and polypharmacy are evolving in the Nepalese context and the extent of their use among older people in the clinical practice of Nepal might surpass that in Western nations, and their distribution may vary across different states in Nepal. This scoping review aimed to find the prevalence of PIM and polypharmacy among older people in lower-middle-income countries like Nepal.

## Materials and Methods

### Protocol and registration

The protocol of this scoping review was registered in the Open Science Framework (https://doi.org/10.17605/OSF.IO/W6S8E) and was conducted in accordance with the Joanna Briggs Institute (JBI) Scoping review framework ^(20)^.

### Population, concept, and context

A systematic search was done using predefined population, concept, and context format search terms. The older adults ≥60 years (population), the PIM and polypharmacy (concept), and the context―Nepal―are the essential components of this review.

### Inclusion and exclusion criteria

Studies conducted in Nepal on patients aged ≥60 years with an assessment of PIM using any explicit criteria (tools) were included. According to the Senior Citizens Acts of Nepal, senior citizens are those who have reached the age of ≥60 years ^(8), (19)^. Hence, the elderly aged ≥60 years are selected for the study. We excluded the studies that did not include the prevalence of PIMs, studies that used implicit techniques to identify PIM, and studies that included patients under the age of 60 years. Systematic reviews and meta-analyses were also excluded to provide direct evidence on the topic and ensure thorough mapping of available research avoiding the duplication and overrepresentation of specific studies.

### Search strategy

Studies were searched using electronic databases: PubMed, Embase, and Google Scholar. Search terms were obtained from concepts of the research question and keywords of relevant articles. Then, a systematic approach for finding relevant articles was applied using a combination of Medical Subject Headings and keywords along with the incorporation of Boolean operators, truncation, and field tags (Table 1). The search was limited to include original studies that were published in the English language from January 1, 2011 to December 15, 2024.

**Table 1.** Search Terms and Strategies for PubMed and Google Scholar.

Database	Search term and strategy
Google Scholar	(older OR elderly OR senior OR aged OR frail OR geriatric OR beers OR stopp OR start) AND (‘potentially inappropriate medication’ OR ‘Deprescribing’ OR ‘Inappropriate prescribing’ OR ‘polypharmacy’) AND (‘nepal’)
PubMed	1.Strategy for Population: (((((“Aged”[Mesh]) OR “Frail Elderly”[Mesh])) OR (elderly OR elder OR geriatric OR “elderly people” OR “elderly person” OR “elderly persons” OR “old people” OR senior OR seniors OR “older adults” OR “older adult”)))
	2.Strategy for Concept: (((((((“Potentially Inappropriate Medication List”[Mesh]) OR “Deprescriptions”[Mesh]) OR “Inappropriate Prescribing”[Mesh]) OR “Polypharmacy”[Mesh])) OR (deprescrib* OR STOPP[tiab] OR Beers[tiab] OR PIM[tiab] OR “potentially inappropriate medications” OR polypharmacy)) OR (discontinu* OR inappropriate OR “high risk” OR unnecessary OR optimiz* OR optimis* OR rationaliz* OR rationalis* OR futility OR futile OR non-essential AND medic*))) OR (((prescription pattern[Title/Abstract]) OR (prescription error[Title/Abstract])) OR (drug utilisation pattern[Title/Abstract])) OR (“potentially inappropriate medication”[Title/Abstract])
	3.Strategy for Context: (((“western nepal”) OR (“eastern nepal”)) OR (“central nepal”)) OR (Nepal)
	4.Final search strategy for PubMed: Strategy 1 AND Strategy 2 ANDStrategy 3
Embase	1.Strategy for Population: ‘older adults’/exp OR ‘older adults’ OR aged OR ‘frail elderly’/exp OR ‘frail elderly’
	2.Strategy for Concept: ‘potentially inappropriate medication’/exp OR ‘potentially inappropriate medication’ OR deprescription OR ‘polypharmacy’/exp OR polypharmacy OR ‘unnecessary prescribing’ OR ‘beers criteria’ OR ‘stopp start criteria’/exp OR ‘stopp start criteria’
	3.Strategy for context: ‘nepal’/exp OR nepal
	4.Final search strategy for PubMed: Strategy 1 AND Strategy 2 AND Strategy 3

### Study selection

All the included studies were screened using Covidence (https://app.covidence.org/), a web-based platform that streamlines the review process, initially for title and abstract and subsequently for those selected for the full texts. The articles were initially reviewed by one reviewer (SS) followed by both reviewers (AP and AK) and conflicts were resolved by all three reviewers (SS, AP, AK) after a thorough discussion. Reference lists of these articles were scanned to identify additional relevant articles.

### Data extraction and analysis

A specially designed data extraction form was used to include information from each selected study on author details, publication year, geography, study design, study setting, population characteristics (age, sex), sample size, primary findings (prevalence of PIMs, explicit tool used and list of frequently prescribed PIMs) and secondary finding (prevalence of polypharmacy). Data was extracted by one reviewer (AK) and further checked, revised, and confirmed by the other two reviewers (AP, SS). Data from individual studies were entered in MS Excel and the statistical formula for weighted average in MS Excel was used to derive the average value for the prevalence of PIMs and polypharmacy ^(17), (18), (19), (20), (21), (22), (23)^. However, due to lack of range, standard deviation, standard error, and unavailability of raw data, the confidence interval could not be derived from prevalence % and sample size only.

## Results

### Study selection

**Figure 1.**
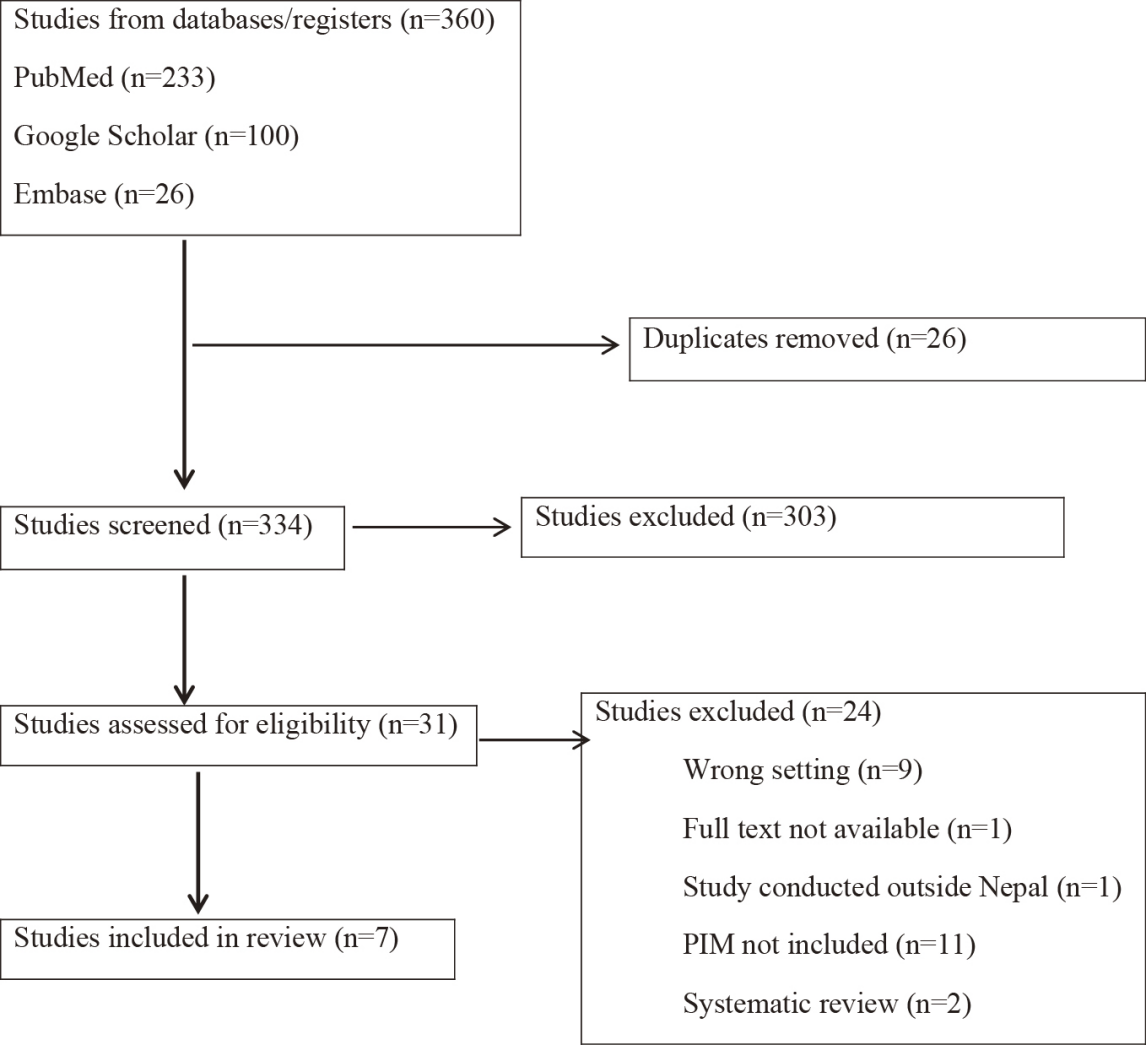
PRISMA flow chart for study selection. PRISMA: Preferred Reporting Items for Systematic reviews and Meta-Analyses.

A total of 360 studies conducted in Nepal were identified (Figure 1). After removing 26 duplicates, 334 studies were screened for title and abstract which led to 31 studies for full-text screening. Finally, seven studies that met all the inclusion criteria were included in this review ^(17), (18), (19), (20), (21), (22), (23)^. The studies included are summarized in Table 2.

**Table 2.** An Overview of Included Studies ^(17), (18), (19), (20), (21), (22), (23)^.

Author (Year)	Setting, location	Duration	Study design	Sample size (age)	Primary Findings			Secondary findings
					PIM%	Tools used	Frequently prescribed PIM (%)	Polypharmacy %
Shrestha S et al. (2024)	Private hospital, Biratnagar	6 months (March 2022 to August 2022)	Retrospective cross-sectional study	n=225 (≥65 years)	18.05%	Beers 2015 updated criteria	Proton pump inhibitors (39.09%), NSAIDs (25.49%)	NA
Chaudhary SK. (2021)	Community pharmacy, Bhaktapur	3 months (December 2020 to March 2021)	Cross-sectional study	n=114 (>60 years)	38.50% as per STOPP criteria and 8.70% as per START criteria	STOPP and START criteria	NSAIDs (25.3%)	44.70% patients 5 to 7 drugs per prescription followed by 8 to 9 drugs per prescription in 18.40%
Giri SA et al. (2020)	Teaching Hospital, Pokhara	4 months (August 2018 to November 2018)	A prospective cross-sectional study	n=403 (≥65 years)	21.60%	Beers 2015 updated criteria	NSAIDs (44%), Anticholinergics (18%)	49.13% (high-level polypharmacy 4.96%)
Rijal S et al. (2019)	Zonal Hospital, Biratnagar	4 months (May 2018 to August 2018)	A prospective cross- sectional study	n=200 (≥60 years)	14%	Beers criteria 2012	Benzodiazepines (46.43%), Antiemetic- Dopamine agonist (28.57%)	62%
Sah AK et al. (2017)	Teaching Hospital, Bharatpur	12 months (November 2012 to October 2013)	A prospective observational analysis	n=869 (≥65 years)	34.30%	Beers criteria	Antihistaminics (12%), Anticholinergics (10.43%)	NA
Basnet S et al. (2016)	Teaching Hospital, Bharatpur	2 months (January and February 2014)	Single-center retrospective cross- sectional and observational study	n=225 (≥65 years)	7.21%	Beers criteria	Benzodiazepines (25.56%). NSAIDs (16.54%)	86.66% (33.30% high level polypharmacy, >10 drugs)
Sapkota S et al. (2011)	Teaching Hospital, Kathmandu	3 months (April to June 2010)	A retrospective study	n=305 (≥65 years)	53%	Beers criteria	Benzodiazepines (32.42%), NSAIDs (29.67%)	NA

Abbreviations: START, Screening tool to alert doctors to right treatment; STOPP, Screening tool of older people’s potentially inappropriate prescription; PIM, potentially inappropriate medications; NA, not available Definition: PIM, medicines prescribed to a given patient for whom the risks outweigh the benefits ^(6)^; Polypharmacy, use of five or more medicines ^(10)^

### Study characteristics

The study characteristics are presented in Table 1. Out of seven included studies, all of them reported the prevalence of PIMs using explicit tools and four of them reported the prevalence of polypharmacy ^(21), (22), (23), (24), (25), (26), (27)^. The included studies comprised a total of 2341 participants with most being men (60.8%). Five studies included elderly patients aged ≥65 years whereas two studies included elderly patients aged ≥60 years. All the included studies were cross-sectional, retrospective (n = 3) and prospective (n = 4) ^(21), (22), (23), (24), (25), (26), (27)^. There was wide regional representation from Nepal. Studies mostly represented central Nepal (n = 4) (Bharatpur, n = 2; Bhaktapur, n = 1; Kathmandu, n = 1) ^(21), (22), (24), (26)^ followed by two studies from eastern Nepal (Biratnagar, n = 2) ^(24), (25)^ and one study from western Nepal (Kaski) ^(23)^.

### Prevalence of PIMs

The prevalence of PIMs in Nepal was 28.5% which varied from the eastern region (14%-18.05%) to the central region (7.21%-53%), to the western region (21.60%) ^(21), (22), (23), (24), (25), (26), (27)^. Six studies used Beers criteria while one study used both STOPP and START criteria to assess PIMs. The most commonly reported PIMs were benzodiazepines (25.56%-46.43%), non-steroidal anti-inflammatory drugs (16.54%-44%), proton pump inhibitors (39.09%), anticholinergics (10.43%-18%), and dopamine agonist as antiemetic (28.57%) ^(21), (22), (23), (24), (25), (26), (27)^.

### Prevalence of polypharmacy

The prevalence of polypharmacy was 39.48%, which was only reported in four studies with a minimum prevalence of 44.7%-86.66% in central Nepal, to 49.13% in western Nepal, and 62% in eastern Nepal ^(21), (22), (23), (27)^.

## Discussion

To the best of our knowledge, this is the first scoping review to assess the prevalence of PIM use and polypharmacy among older people in Nepal. Selected studies were from three regions―east, west, and central Nepal―which featured distinct demographic and socio-economic characteristics. Kathmandu, Bhaktapur, and Bharatpur are from central Nepal with diverse ethnic groups such as Newars, Brahmins, and Chhetris, and better infrastructure and opportunities for employment and health facilities ^(8), (19)^. Eastern Nepal has people of Limbus, Rais, and Madhesi ethnicity with significant industrial and agricultural activities with better connectivity and remittance income but still less developed than central Nepal ^(8), (19)^. Western Nepal is the least developed with people of all ethnicities but is the least populated, mostly hilly terrain, lower literacy rates, and relies primarily on agriculture and tourism ^(8), (19)^.

### PIMs

This study analyzed data from seven studies that involved 2341 adults with wider variation in the prevalence of PIMs (28.5%). A systematic review from Malaysia reported a similar prevalence of PIMs, that is, 28.9% (95% confidence interval: 25.4-32.3) ^(16)^. Studies conducted in India, a similar geographical setting to Nepal, reported the a prevalence of PIMs of 8.4%, 17.3%, 34%, and 53.5%, and other studies from Malaysia also reported a prevalence of PIMs from 13.6% to 34.9% which showed wide variations ^(28), (29), (30), (31), (32), (33), (34), (35)^. This variation may be due to differences in study populations, comorbidities, disparities in prescribing practices, and health care provider’s knowledge, experience, and adherence to clinical guidelines. In addition, there was a variation in the explicit criteria used to evaluate PIMs.

Five studies selected in this review used the Beers criteria, which is a well-known and one of the most commonly used explicit criteria to identify PIMs in the geriatric population ^(36), (37)^. These explicit tools emphasize the significance of defined criteria for thorough assessments and address the difficulty of researching PIMs among older people ^(37)^. The Beers criteria are user-friendly, with clear and concise recommendations that enhance their practical applicability, making them accessible to healthcare professionals with varying levels of expertise and resources in developing countries ^(6), (37)^. However, none of the studies conducted in Nepal used Asian PIMs criteria, which were developed using the Delphi method ^(4), (38)^. This explicit tool provides a foundation for optimizing medicine use based on local healthcare requirements and resources by offering a flexible framework that may be tailored to the demands of poor countries ^(4), (10), (15), (38)^. Older people are more prone to have multiple morbidities, which increases their chance of using the wrong drugs ^(39)^.

The most frequently prescribed inappropriate drugs were benzodiazepines, non-steroidal anti-inflammatory drugs, anticholinergics, antihistamines, and antiemetics which are similar to those reported by various studies which were in congruence with findings from India, Pakistan, Sri Lanka, China and Malaysia ^(4), (30), (33), (34), (35), (36)^. Corrective prescription trends may be difficult to address if strong regulatory frameworks are lacking and comprehensive drug management techniques are not implemented well.

### Polypharmacy

Out of seven studies, only four studies reported polypharmacy and the prevalence of polypharmacy was 39.48% which lies within the range reported in several studies (39.4%-66.2%) ^(4), (30), (33), (34), (35), (36), (38), (39), (40)^. In the Nepali context, polypharmacy is an increasing trend that may be attributed to the increasing trend of chronic diseases among the aging population, leading to the necessity for multiple medications to manage various comorbidities. Moreover, limited access to health care and a shortage of healthcare professionals may contribute to suboptimal monitoring and coordination of medication regimens. Additionally, patient factors such as low health literacy, cultural beliefs, and a tendency to seek care from multiple healthcare providers to get faster recovery might lead to fragmented care and the inadvertent accumulation of medications ^(18), (29)^. Polypharmacy is a complex topic with potential differences in appropriateness and consequences between healthier and diseased individuals. In developing countries like Nepal, the influence of pharmaceutical marketing on prescribing practices cannot be ruled out for causing high prescription rates. For the safe and effective use of medications, these factors must be addressed, and polypharmacy should be justified and kept at a minimum, particularly in elderly or frail persons ^(41), (42)^. Various approaches have been suggested to mitigate polypharmacy, including deprescribing, medication reconciliation practices, diminishing the utilization of unnecessary and unintentional medications raising awareness on avoiding dichotomy practices, and minimizing medication overuse ^(43)^.

### Polypharmacy and PIMs

Polypharmacy appears to be the most obvious risk factor of PIMs ^(44)^. More recently, it has been demonstrated that deprescribing helps elderly people experience reduced PIMs and polypharmacy ^(45), (46)^. The term “deprescribing.” which refers to the rationalization of medications, has drawn a lot of interest lately. It is described as the systematic method of determining which medications to stop taking when the risks of doing so outweigh the benefits when taking into account the patient’s values, preferences, life expectancy, goals for their care, and present functioning level. None of the studies that we analyzed included any information about medication withdrawal ^(47)^. Thus, it is important to cease prescribing unnecessary medications inappropriately to decrease polypharmacy. A successful prescribing continuum should include deprescribing and considering the duration of treatment, when to stop treatment, and how to do it, both during the initial phase of treatment and as it continues ^(47)^. Medication removed from the overall care should have a plan in place ^(48)^. These results highlight the critical need for focused interventions and legislative changes to address the widespread problems of polypharmacy and PIMs among Nepal’s older population. In Nepal, one in two elderly seniors receiving ambulatory care would agree to have one or more of their prescription drugs discontinued ^(48), (49), (50)^. It is imperative to motivate medical professionals to weigh the advantages and disadvantages of prescription drugs for elderly patients. To reduce the use of PIMs, initiatives such as collaborative prescriber-pharmacist reviews of medications, shared decision-making, regular medication safety training, and the creation of senior patient-centered drug use education programs should be encouraged ^(46), (51)^. Addressing the increasing trend of PIMs and polypharmacy calls for a multifaceted approach which includes strengthening healthcare infrastructure, improving health literacy, enhancing regulatory oversight, and promoting evidence-based prescribing and deprescribing practices ^(48), (49), (51)^.

This scoping review was the first of its kind conducted in Nepal regarding the evidence synthesis on PIM. This scoping review sheds important light on the medication trends affecting this vulnerable population by examining the frequency of PIMs and polypharmacy among older adults in Nepal. One of the review’s strengths is how thoroughly it examined the body of literature, covering a range of study designs and approaches. This method makes it possible to comprehend PIMs and polypharmacy prevalence in Nepal’s elderly population in a comprehensive manner. Furthermore, the review aids in pointing out gaps in the existing literature, opening the door for further studies to fill up these knowledge gaps. However, variations in sample sizes, the caliber of the data that are accessible, and the limited database could compromise the findings’ comparability.

### Conclusions

The prevalence of PIMs and polypharmacy among older Nepalese adults was relatively high. This scoping review is an essential starting point for comprehending the drug landscape in the geriatric population in Nepal. The average prevalence of PIMs in published studies highlights the significant negative effects of inappropriate medication practices on the health of the elderly population. Furthermore, though polypharmacy rates are a secondary result, they highlight a serious issue in the elderly patients under study. These findings highlight the need for urgent steps to develop a PIMs criterion for older adults in Nepal and deprescribing criteria that suit the Nepali context. In addition, it necessitates promoting rational prescribing and prioritizing comprehensive medication reviews to reduce medication-related problems in older adults and to conduct a deprescribing randomized controlled trial in older adults.

## Article Information

### Conflicts of Interest

None

### Author Contributions

Anjan Khadka and Shakti Shrestha conceived the review topic. All authors reviewed articles and resolved the conflicts. Anjan Khadka contributed to the data extraction which was checked, revised and confirmed by Shakti Shrestha and Arjun Poudel. All authors critically reviewed and revised the manuscript and approved the final version for submission. Shakti Shrestha supervised the overall review process.

### Approval by Institutional Review Board (IRB)

The studies used in this scoping review are published in several medical journals. Ethics approval was not required for scoping review.
